# Memory, Emotion, and Age: The Work of Kinugawa et al. (2013)

**DOI:** 10.3389/fpsyt.2014.00058

**Published:** 2014-05-27

**Authors:** Hans J. Markowitsch

**Affiliations:** ^1^Department of Physiological Psychology, University of Bielefeld, Bielefeld, Germany; ^2^Center of Excellence Cognitive Interaction Technology, University of Bielefeld, Bielefeld, Germany

**Keywords:** empathy, autobiography, time, neuronal changes, intellect

Memory is the key to our life. Already in 1870, the famous physiologist Hering (1) stated in a booklet that memory “connects innumerable single phenomena into a whole, and just as the body would be scattered like dust in countless atoms if the attraction of matter did not hold it together so consciousness – without the connecting power of memory – would fall apart in as many fragments as it contains moments” (p. 12; my translation). With this statement, he emphasized that healthy human beings are able to mentally travel in time and that in their presence they can learn from their past to find optimal solutions for their future. It is known that this capacity needs time, brain maturation, and the establishment of an autonomous self (including theory of mind abilities and the ability to show empathy) in order to develop and persist (2). Adults consequently cannot consciously remember events that had occurred prior to the age of 3–4 years (3–6). On the other hand, in dementia the ability to travel back in time deteriorates and then gets lost totally (7). The deterioration of episodic–autobiographical memory [cf. the Figure in Ref. (8), or Figure 3 in Ref. (9)] is accompanied by a reduction in emotional colorization of retrieved events – the descriptions become more fact-like, are less detailed, and are reproduced without corresponding affect (10) (Figure 1). The reduced emotional impact on behavior in old people extends to a number of everyday situations (11) and may lead to retrieval deficits especially in situations guiding retrieval.

**Figure 1:**
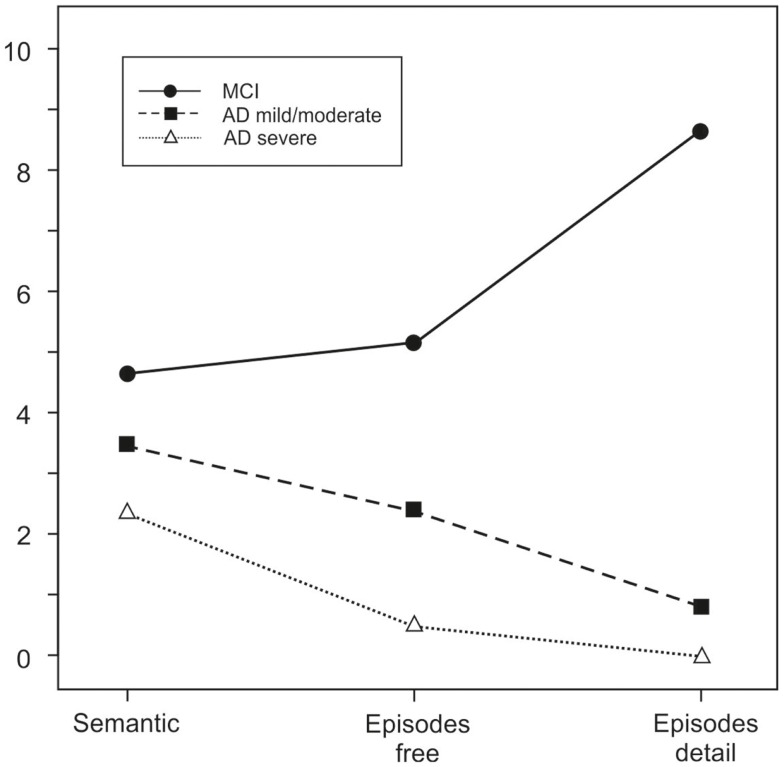
**Production of semantic and episodic–autobiographical details in patients with mild cognitive impairment (MCI), mild to moderate Alzheimer’s disease (AD mild/moderate), and severe Alzheimer’s disease (AD severe)**. Data from Seidl et al. (10).

The interaction between memory and emotion across the life span was the topic of a nicely designed experiment by Kinugawa et al. (12). A “what, where, and when” paradigm was used that allowed measuring content and temporal and spatial context of an event by combining the presentation of visual scenes with a context story with an emotionally arousing content. Participants were divided into three groups of 17, 16, and 8 volunteers with mean ages of 27, 55, and 79 years, and tested with this paradigm. The principal outcome was that the old compared to the young group was poorer in recall and had in addition a lower working memory performance and less anxiety as measured by state and trait anxiety scales. These results add to a number of related ones, demonstrating that older people recruit more brain regions to perform a given task (13), have a less sharp differentiation between explicit and implicit memory processing (14), and perceive life processes as speeding up with age (15). Furthermore, it was found that old people have a higher tendency to forget negative emotional memories, compared to positive or neutral ones (16).

Already in the year 2005, Drachman (17) in an editorial highlighted the manifold brain changes in the elderly (e.g., neuronal loss, white matter loss, volume shrinkage, sulcal widening, reduction of synaptic density), which probably account for the intellectual decline found with increasing age. Drachman wrote: “Using the standard age correction for the Wechsler Adult Intelligence Scale (WAIS), to obtain an IQ score of 100 at age 75, one need to answer only half as many questions correctly as at age 21!” (p. 2005). Though the neuronal and intellectual decline may not be universal (18), it still affects the great majority of old people (19). Conventional mental training may be a much less effective counteractive treatment (20) than physical exercise (21, 22). The new episodic memory task proposed by Kinugawa et al. might nevertheless be apt to induce new attempts not only to assess, but also to train memory in older people. The authors themselves suggest a number of possible approaches for future research in this direction.

## Conflict of Interest Statement

The author declares that the research was conducted in the absence of any commercial or financial relationships that could be construed as a potential conflict of interest.
